# Achilles Tendon Rupture: A Bibliometric Study

**DOI:** 10.7759/cureus.87659

**Published:** 2025-07-10

**Authors:** Jacob Markel, Jacob T Williamson, Jordan Pamplin, Victor Anciano

**Affiliations:** 1 Orthopedic Surgery, Grand Rapids Orthopaedic Foot and Ankle Fellowship, Grand Rapids, USA; 2 Orthopedic Surgery, University of Louisville Health, Louisville, USA

**Keywords:** achilles tendon, achilles tendon rupture, bibliometric analysis, research trends, tendon rupture

## Abstract

Achilles tendon rupture is a frequently addressed subject in foot and ankle research. A bibliometric analysis of the existing literature may provide valuable insights for surgeons and others involved in the diagnosis, treatment, and research of Achilles tendon ruptures. The purpose of this study was to identify and analyze the most-cited research articles regarding Achilles tendon rupture.

Using the Web of Science Database,* *over 1500 publications were identified. The search was refined to include only original articles, resulting in the inclusion of 1,341 articles. Co-authorship between authors was analyzed using a readily available online citation manager software. The 50 most-cited articles were exported into Microsoft Excel for analysis of publication year, journal, authors, and institution of publication.

Of the 1,341 articles published between 1948 and 2023, a notable concentration appeared in 2021 and 2022, with 117 and 113 publications, respectively. The 50 most-cited articles were published between 1974 and 2017, and were cited an average of 166 times, ranging from 79 to 672 citations. The American Journal of Sports Medicine was the most prolific journal with 10 of the top 50 most-cited articles.

The substantial volume of research on Achilles tendon rupture can make it challenging for surgeons and other stakeholders to navigate the relevant literature. Bibliometric analyses such as this provide a structured overview and help identify the most significant contributions in the field.

## Introduction and background

The Achilles tendon was first described as a distinct anatomical structure in 1693 by the anatomist Philip Verheyen. Prior to this, it had been referred to as the ‘tendo magnus’ or, more modestly, the ‘chorda Hippocratis,’ a term attributed to Hippocrates [1]. Irrespective of its nomenclature, the first described Achilles tendon rupture was documented in 1575 by Ambroise Paré [2]. Since its first description, the debilitating nature of this injury has remained self-evident. As a result, Achilles tendon rupture and its management have become some of the most extensively studied topics in the foot and ankle literature.

Presently, a database search for “Achilles rupture” yields over 4,000 results, with results ranging from the years 1852 to 2023. This number appears staggering, and all rationale would indicate that this mounting body of research will continue to grow, in the same exponential pattern demonstrated by scientific literature as a whole [3]. To help navigate this expanding body of research, bibliometric analyses have been widely employed across various fields of medicine [4-6]. Bibliometrics is the application of mathematical methods to evaluate published documents [7]. This is commonly used to determine the most influential articles available in a particular field of study. Such application has become commonplace in foot and ankle surgery, with analyses of the most cited articles in hallux rigidus, hallux valgus, ankle arthroplasty, and arthroscopy [8-12]. To date, there exists no such analysis of Achilles tendon rupture and its management.

As the body of research surrounding Achilles tendon rupture and management expands, challenges in navigating this landscape of literature will arise. An analysis of the most highly cited articles in Achilles tendon research may offer valuable insights for surgeons, trainees, and other interested professionals. This would also provide guidance as to the most influential papers within the topic of Achilles tendon rupture. The purpose of this study was to identify and analyze the most cited and influential research articles regarding Achilles tendon rupture. The abstract for this manuscript was previously presented at the Annual AOFAS meeting in 2024.

## Review

Methods

**Figure 1:**
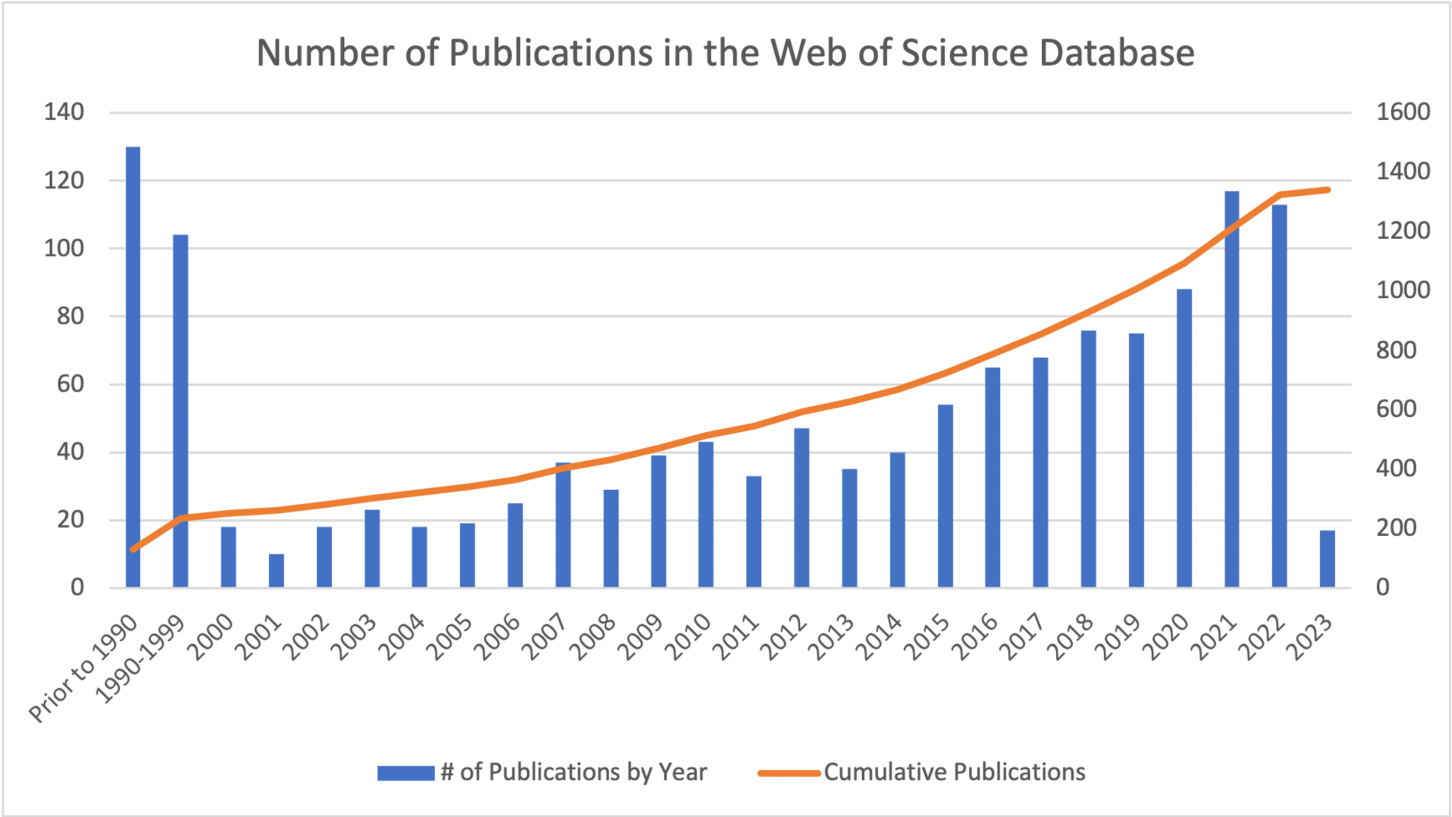
Number of publications in the Web of Science database

The Web of Science Database was queried with the search terms Achilles Tendon Rupture OR Achilles Tendon Tear OR Achilles Tendon Surgery OR Achilles Tendon Repair OR Achilles Tendon Reconstruction. This initially identified over 1500 publications. The search was then refined to include only original articles. This resulted in a total of 1,341 articles included in the final analysis (Figure 1).

The identified articles were then sorted by citation number within the Web of Science Database, and the top 50 most-cited articles were exported into Microsoft Excel (Microsoft Corporation, Redmond, WA, USA) for complete analysis of publication year, journal, authors, impact factor, etc.

Results

A total of 1341 original articles were identified and included in the final analysis, which were published between 1948 and 2023. The number of articles published per year is shown in Figure 1. There was a general trend of increasing publication over time, with the highest number of articles published in 2020, 2021, and 2022, totaling 88, 117, and 113 articles, respectively. Meanwhile, 130 articles were published in the 42 years prior to 1990, and 104 articles were published from 1990-1999.

The 50 most-cited articles are shown in Table 1. All 50 articles were published between 1974 and 2017, and were cited an average of 166 ± 111 times, ranging from 79 to 672 total citations. The study by Young et al., published in the Journal of Orthopaedic Research in 1998, was the most-cited article overall with 672 citations [13]. This article was also the most-cited publication in the past 10 years, with 64 citations since 2013. The top 10 most-cited articles since 2013 are shown in Table 2.

**Table 1:** Top 50 most cited articles

Rank	Article Title	Authors	Year	Citations
1	Use Of Mesenchymal Stem Cells In A Collagen Matrix For Achilles Tendon Repair	Young et al. [13]	1998	672
2	Operative Versus Nonoperative Treatment Of Achilles-Tendon Rupture - A Prospective Randomized Study And Review Of The Literature	Cetti et al. [16]	1993	383
3	The Use Of Xenogeneic Small-Intestinal Submucosa As A Biomaterial For Achilles-Tendon Repair In A Dog-Model	Badylak et al. [15]	1995	381
4	Surgical And Non-Surgical Treatment Of Achilles-Tendon Rupture - A Prospective Randomized Study	Nistor [18]	1981	348
5	Cumulative Incidence Of Achilles Tendon Rupture And Tendinopathy In Male Former Elite Athletes	Kujala et al. [19]	2005	311
6	Acute Achilles Tendon Rupture: A Randomized, Controlled Study Comparing Surgical And Nonsurgical Treatments Using Validated Outcome Measures	Nilsson-Helander et al. [20]	2010	280
7	Surgical Versus Nonsurgical Treatment Of Acute Achilles Tendon Rupture: A Meta-Analysis Of Randomized Trials	Soroceanu et al. [17]	2012	267
8	Incidence Of Achilles Tendon Rupture	Leppilahti et al. [21]	1996	263
9	The Role Of Recreational Sport Activity In Achilles-Tendon Rupture - A Clinical, Pathoanatomical, And Sociological-Study Of 292 Cases	Jozsa et al. [22]	1989	261
10	Changing Incidence Of Achilles Tendon Rupture In Scotland, A 15-Year Study	Maffulli et al. [23]	1999	254
11	Platelet Concentrate Injection Improves Achilles Tendon Repair In Rats	Aspenberg et al. [24]	2004	237
12	Repair Of Chronic Achilles Tendon Rupture With Flexor Hallucis Longus Tendon Transfer	Wapner et al. [25]	1993	206
13	Degradation And Remodeling Of Small Intestinal Submucosa In Canine Achilles Tendon Repair	Gilbert et al. [26]	2007	205
14	Increasing Incidence Of Acute Achilles Tendon Rupture And A Noticeable Decline In Surgical Treatment From 1994 To 2013. A Nationwide Registry Study Of 33,160 Patients	Ganestam et al. [27]	2016	185
15	Structural Achilles Tendon Properties In Athletes Subjected To Different Exercise Modes And In Achilles Tendon Rupture Patients	Kongsgaard et al. [28]	2005	178
16	Achilles Tendon Rupture: A Review Of Etiology, Population, Anatomy, Risk Factors, And Injury Prevention	Hess [29]	2010	169
16	Total Achilles Tendon Rupture - A Review	Leppilahti et al. [30]	1998	169
18	Increasing Incidence Of Achilles Tendon Rupture	Moller et al. [31]	1996	162
19	Major Functional Deficits Persist 2 Years After Acute Achilles Tendon Rupture	Olsson et al. [32]	2011	159
20	Extracellular Matrix Scaffolds Are Repopulated By Bone Marrow-Derived Cells In A Mouse Model Of Achilles Tendon Reconstruction	Zantop et al. [33]	2006	154
21	Deficits In Heel-Rise Height And Achilles Tendon Elongation Occur In Patients Recovering From An Achilles Tendon Rupture	Silbernagel et al. [34]	2012	148
22	The Epidemiology Of Achilles Tendon Rupture In A Danish County	Houshian et al. [35]	1998	147
23	Increased Risk Of Achilles Tendon Rupture With Quinolone Antibacterial Use, Especially In Elderly Patients Taking Oral Corticosteroids	Van Der Linden et al. [36]	2003	145
24	Acute Achilles Tendon Rupture - Minimally Invasive Surgery Versus Nonoperative Treatment With Immediate Full Weightbearing - Randomized Controlled Trial	Metz et al. [37]	2008	134
25	Collagens, Proteoglycans, Mmp-2, Mmp-9 And Timps In Human Achilles Tendon Rupture	Karousou et al. [38]	2008	128
26	Rerupture And Deep Infection Following Treatment Of Total Achilles Tendon Rupture	Pajala et al. [39]	2002	121
27	Achilles-Tendon Rupture Following Steroid Injection - Report Of 3 Cases	Kleinman [40]	1983	120
28	A New Measurement Of Heel-Rise Endurance With The Ability To Detect Functional Deficits In Patients With Achilles Tendon Rupture	Silbernagel et al. [41]	2010	119
29	Prolonged Thromboprophylaxis With Dalteparin After Surgical Treatment Of Achilles Tendon Rupture: A Randomized, Placebo-Controlled Study	Lapidus et al. [42]	2007	112
30	Operative Versus Nonoperative Treatment For Acute Achilles Tendon Rupture: A Meta-Analysis Based On Current Evidence	Jiang et al. [43]	2012	111
30	Open Versus Percutaneous Repair In The Treatment Of Acute Achilles Tendon Rupture: A Randomized Prospective Study	Gigante et al. [44]	2008	111
32	Weakness In End-Range Plantar Flexion After Achilles Tendon Repair	Mullaney et al. [45]	2006	104
33	Comparison Of Conservative And Operative Treatment Of Achilles Tendon Rupture	Jacobs et al. [46]	1978	102
34	Achilles Tendon Rupture - Treatment And Complications: A Systematic Review	Holm et al. [47]	2015	93
35	Functional Treatment Of Acute Achilles-Tendon Rupture - 2-Years Follow-Up Results Of A Prospective Randomized Study	Thermann et al. [48]	1995	92
36	Functional Postoperative Treatment Of Achilles-Tendon Repair	Carter et al.[49]	1992	90
37	Surgical Treatment Versus Conservative Management For Acute Achilles Tendon Rupture: A Systematic Review And Meta-Analysis Of Randomized Controlled Trials	Deng et al. [50]	2017	88
37	Wound Complications After Open Achilles Tendon Repair - An Analysis Of Risk Factors	Bruggeman et al. [51]	2004	88
37	Traumatic Rupture Of Achilles Tendon - Reconstruction By Transplant And Graft Using Lateral Peroneus Brevis	Teuffer AP [52]	1974	88
40	Avoiding Sural Nerve Injuries During Percutaneous Achilles Tendon Repair	Majewski et al. [53]	2006	87
41	Nonoperative Dynamic Treatment Of Acute Achilles Tendon Rupture: The Influence Of Early Weight-Bearing On Clinical Outcome A Blinded, Randomized Controlled Trial	Barfod et al. [54]	2014	85
41	The Increasing Incidence And Difference In Sex Distribution Of Achilles Tendon Rupture In Finland In 1987-1999	Nyyssonen et al. [55]	2008	85
41	Early Full Weightbearing And Functional Treatment After Surgical Repair Of Acute Achilles Tendon Rupture	Speck et al. [56]	1998	85
44	The Strength Of Achilles-Tendon Repair - An In-Vitro Study Of The Biomechanical Behavior In Human Cadaver Tendons	Watson et al. [57]	1995	84
45	Is Percutaneous Repair Better Than Open Repair In Acute Achilles Tendon Rupture?	Henriquez et al. [58]	2012	83
45	Operative Versus Nonoperative Management Of Acute Achilles Tendon Rupture - Expected-Value Decision Analysis	Kocher et al. [59]	2002	83
47	Early Nerve Regeneration After Achilles Tendon Rupture - A Prerequisite For Healing? A Study In The Rat	Ackermann et al. [60]	2002	81
48	High Incidence Of Deep Venous Thrombosis After Achilles Tendon Rupture: A Prospective Study	Nilsson-Helander et al. [61]	2009	80
48	Calf Muscle Function After Achilles Tendon Rupture - A Prospective, Randomised Study Comparing Surgical And Non-Surgical Treatment	Moller et al. [62]	2002	80
50	Epidemiology And Outcomes Of Acute Achilles Tendon Rupture With Operative Or Nonoperative Treatment Using An Identical Functional Bracing Protocol	Gwynne-Jones et al. [63]	2011	79

**Table 2:** Top 10 most-cited articles sorted by citations since 2013

Rank	Article Title	Authors	Year	Citations
1	Use Of Mesenchymal Stem Cells In A Collagen Matrix For Achilles Tendon Repair	Young et al. [13]	1998	64
2	Surgical Versus Nonsurgical Treatment Of Acute Achilles Tendon Rupture: A Meta-Analysis Of Randomized Trials	Soroceanu et al. [17]	2012	51
3	Cumulative Incidence Of Achilles Tendon Rupture And Tendinopathy In Male Former Elite Athletes	Kujala et al. [19]	2005	49
4	Achilles Tendon Rupture - Treatment And Complications: A Systematic Review	Holm et al. [47]	2015	43
5	The Use Of Xenogeneic Small-Intestinal Submucosa As A Biomaterial For Achilles-Tendon Repair In A Dog-Model	Badylak et al. [15]	1995	38
6	Operative Versus Nonoperative Treatment For Acute Achilles Tendon Rupture: A Meta-Analysis Based On Current Evidence	Jiang et al. [43]	2012	37
7	Structural Achilles Tendon Properties In Athletes Subjected To Different Exercise Modes And In Achilles Tendon Rupture Patients	Kongsgaard et al. [28]	2005	35
8	Achilles Tendon Rupture: A Review Of Etiology, Population, Anatomy, Risk Factors, And Injury Prevention	Hess [29]	2010	31
9	Degradation And Remodeling Of Small Intestinal Submucosa In Canine Achilles Tendon Repair	Gilbert et al. [26]	2007	30
10	Acute Achilles Tendon Rupture A Randomized, Controlled Study Comparing Surgical And Nonsurgical Treatments Using Validated Outcome Measures	Nilsson-Helander et al. [20]	2010	28

Co-authorship

**Figure 2:**
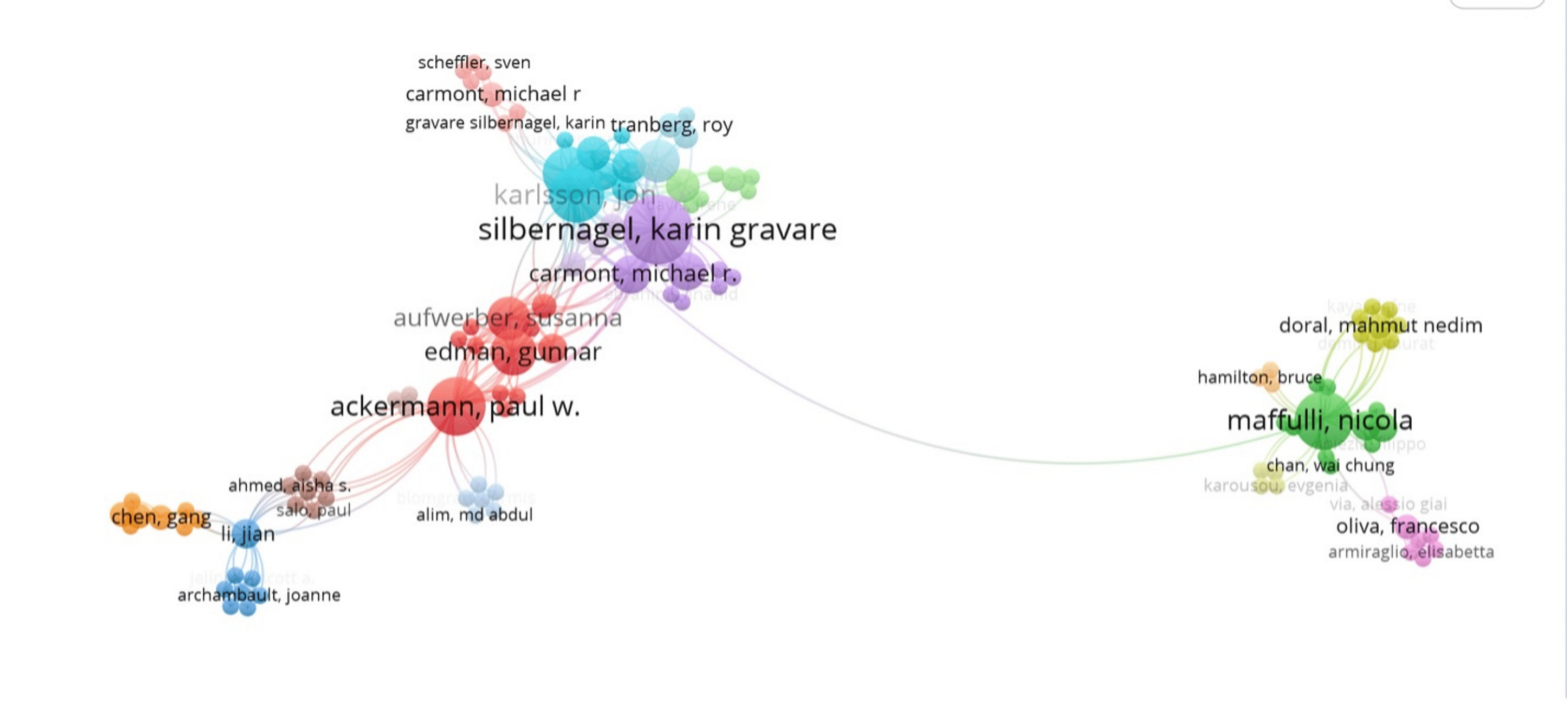
Map demonstrating author relationships

Co-authorship between individual authors was analyzed using VOSviewer (Leiden University, Leiden, The Netherlands), a freely available citation mapping software (Figure 2). Figure 2 provides a map of co-authorship within the 50 most-cited articles analyzed. Analysis indicated that Silbernagel KG, Karlson J, Ackerman P, and Maffulli N were the authors with the most significant contributions to this research on Achilles tendon rupture. Leppilahti J, Nilsson-Helander K, and Silbernagel K were the most prolific primary authors, with 2 publications each.

Country of Origin

The research represented in the top 50 publications originated in 16 different countries and was published by 22 unique medical journals from 6 different countries (Tables 1, 2). The average impact factor of the journals represented in this body of research was 8 ± 5.7 and ranged from 1.5 to 43.2 (Scandinavian Journal of Medicine & Science in Sports, and Archives of Internal Medicine, respectively).

Twenty-two different journals in six unique countries were included, with The American Journal of Sports Medicine contributing the most (11/50). Interestingly, this is different than other foot and ankle bibliometric analyses, which have often shown either the Journal of Bone and Joint Surgery (JBJS) or the Foot and Ankle International (FAI) as the leading journals [9-14]. In this study, six articles were published by JBJS (the second highest contributor), while 3 appeared in FAI. The vast majority of articles were published in the United States (35/50). This was closely followed by Sweden with 10 and Denmark with six. In total, there were contributions from researchers based in 16 unique countries on 5 of the 7 world continents (excluding only Africa and Antarctica). Other bibliometric analyses in the foot and ankle literature have also found the United States to be the most productive country, with similar rates of papers publishedas reported in this study [9-12]. The broad spread of the location of origin of the research included in this study also highlights the interest in this condition worldwide and the increasingly broad landscape of foot and ankle research generally.

Chronology 

The distribution of publishing year of our original search, which included over 1300 publications, can be seen in Figure 1. Although research activity on this topic has steadily increased over the years, newer articles were not necessarily cited more frequently. On the contrary, all of the top 50 most-cited articles were published between 1974 and 2017. Of these, only four have been published in the past ten years. Although older articles have had more time to accumulate citations, this does not necessarily imply that earlier literature is more significant than newer research. To better capture recent research trends, we also analyzed the most-cited articles from the past 10 years (Table 2). While the results were slightly different, the most-cited papers tended to be the most cited in the past 10 years as well. There continues to be an upward trend in research regarding Achilles rupture, which will likely culminate in an increasing number of citations for these more recent publications.

Biologics

The most-cited publication of this study was the article by Young et al., which was published in 1998 in the Journal of Orthopaedic Research and cited 672 times overall [13]. Young et al. assessed the biomechanics of mesenchymal stem cell-seeded implants versus suture material. This article attempts to help guide the operative management of Achilles Rupture. Its results demonstrated that the use of mesenchymal stem cells in the repair of a tendon defect is biomechanically improved when compared to repair with suture alone. This article as helped guide treatment and further research of the use of biologics in the repair of Achilles Tendon ruptures, and is the most-cited article overall and in the past 10 years.

The use of biologics and, more broadly, the optimal operative management in the surgical treatment of Achilles Tendon rupture is an important topic of research. The third most-cited article, written by SF Badylak et al., investigated the use of xenogeneic biomaterial in dogs [15]. Their study indicated that the use of xenogeneic biomaterial resulted in stronger tendons when compared with ruptures treated with traditional suture fixation and ruptures treated non-operatively. This study was cited 381 times. In addition to the two articles by Young et al. and Badylak et al., six other studies in this analysis investigated topics broadly encompassing the use of biologics and/or grafts in the surgical treatment of Achilles rupture, while three other articles investigated differences between traditional open and percutaneous surgical management of Achilles rupture.

Operative and Non-Operative Treatment

Perhaps the most significant topic in the literature on Achilles tendon rupture is the debate between non-operative versus operative management. The second most-cited article overall, Cetti et al., and the second most-cited article since 2013, Soroceanu et al., investigated this subject [16,17]. Ten articles in the top 50 most-cited, including four articles in the top ten, investigated the general topic of operative vs. non-operative treatment in Achilles tendon rupture.

Limitations

There were several limitations of our study. The first being that only one database was used to obtain the articles used in this study. This may limit the available literature, as there is a small possibility that some articles are not included in the database. This study aimed to identify the most-cited articles; however, the number of citations does not necessarily correlate with the quality of those citations. While this study can serve as an initial reference for those researching Achilles tendon ruptures and management, future studies would be beneficial to investigate the significance of these landmark studies.

## Conclusions

The abundance of literature on Achilles tendon rupture and its management has resulted in a substantial body of available research. The purpose of this article was to perform a bibliometric analysis of the current literature to define the top 50 most-cited articles on this topic. The use of biologics is a well-established and extensively studied aspect of Achilles tendon rupture management. Two of the top three most cited articles on Achilles rupture focus on the augmentation of Achilles tendon repair using biologics. Further, six studies within the top fifty discuss the topic more broadly.

The United States continues to be the most productive country contributing to this topic. Overall, there were contributions from authors based in 16 countries. There were 22 journals in six countries included in our review, with the most prevalent being The American Journal of Sports Medicine, followed by The Bone and Joint Surgery and Foot & Ankle International. This is in contrast to previous bibliometric studies on the topic. The debate of non-operative versus operative management of Achilles rupture remains highly contested. Ten articles in the top 50 most-cited, including four articles in the top ten, investigated the general topic of operative vs. non-operative treatment in Achilles tendon rupture. Our study will help surgeons, students, and other stakeholders navigate the relevant literature and identify significant contributions in the field of Achilles tendon rupture.
